# Experiences from multiplex PCR diagnostics of faeces in hospitalised patients: clinical significance of Enteropathogenic *Escherichia coli* (EPEC) and culture negative campylobacter

**DOI:** 10.1186/s12879-019-4271-1

**Published:** 2019-07-17

**Authors:** Jan-Erik Berdal, Benoit Follin-Arbelet, Jørgen Vildershøj Bjørnholt

**Affiliations:** 1 Department of Infectious Diseases, Akershus University Hospital, PO Box 1000, 1478 Lørenskog, Nordbyhagen Norway; 2 Institute of Clinical Medicine, University of Oslo, Oslo, Norway; 3 Present Address: Institute of Oral Biology, University of Oslo, Oslo, Norway; 4 Department of Clinical Microbiology, Oslo University Hospital, Oslo, Norway; 5 Department of Microbiology, Akershus University Hospital, Nordbyhagen, Norway

**Keywords:** Enteropathogenic *Escherichia coli*, Campylobacter, Diagnostic tests, Diarrhoea, Gastroenteritis

## Abstract

**Background:**

In hospitalised patients with diarrhoea a positive campylobacter stool Polymerase Chain Reaction (PCR) test with negative culture results as well as Enteropathogenic *Escherichia coli* (EPEC) positive stool PCRs, challenges the clinician and may lead the unexperienced clinician astray. The aim of the study was to elucidate the clinical significance of positive Campylobacter and/or EPEC test results in hospitalised patients with diarrhoea.

**Methods:**

We conducted a retrospective case-case study. Case groups with 1) EPEC only and 2) EPEC in combination with any other pathogen in the PCR multiplex array, 3) PCR positive/culture negative Campylobacter, and 4) PCR positive/culture positive Campylobacter were compared. Medical records were reviewed and cases classified according to pre-specified clinical criteria as infectious gastroenteritis or non-infectious causes for diarrhoea. We analyzed the association between laboratory findings (the 4 subgroups) and the pre-specified clinical classification. We further sequenced culture negative campylobacter samples and tested EPEC for bundle forming pilus A (bfpA) gene, distinguishing typical from atypical EPEC.

**Results:**

A total of 291 patients were included, 169 were PCR positive for Campylobacter and 122 for EPEC. For both pathogens, co-infections were more common in culture negative/PCR positive samples than in culture positive samples. Clinical characteristics differed significantly in and between groups. Campylobacter culture positive patients had very high prevalence of characteristics of acute infectious gastroenteritis, whereas patients with PCR positive test results only often had an alternative explanation for their diarrhoea. Culture positives were almost exclusively *C. jejuni/coli*, whereas in culture negatives, constituting a third of the total PCR positives, *C. concisus* was the most frequent species. The vast majority of EPEC only positives had documented non-infectious factors that could explain diarrhoea. The EPEC co-infected group mimicked the culture positive campylobacter group, with most patients fulfilling the infectious gastroenteritis criteria.

**Conclusions:**

In hospitalised patients, positive PCR results for campylobacter and EPEC should be interpreted in a clinical context after evaluation of non-infectious diarrhoea associated conditions, and cannot be used as a stand-alone diagnostic tool.

## Background

Diarrhoea in hospitalised patients may have a number of non-infectious causes [1]. The likelihood of discovering gastroenteritis pathogens decreases substantially for diarrhoea occurring in patients hospitalised for ≥3 days [2]. On the other hand, several conditions associated with non-infectious diarrhoea may necessitate hospitalization, and broad differential diagnosis remains for patients with diarrhoea at admission. Due to its ease and speed, multiplex Polymerase Chain Reaction (PCR) testing for gastroenteritis pathogens has largely replaced culture-based identification. However, findings of multiple pathogens in the same sample and discordant PCR and culture results are not uncommon. This may confuse the clinician’s interpretation of test results. At our institution, a large university hospital servicing almost 10% of the Norwegian population, Campylobacter and Enteropathogenic *Escherichia coli* (EPEC) are the two most common fecal pathogens detected by PCR. With the overall aim of elucidating the clinical relevance of positive PCR tests for these pathogens, we investigated the distribution of clear-cut infectious gastroenteritis and non-gastroenteritis medical conditions associated with diarrhoea in all patients testing positive by PCR and/or culture. We further sequenced campylobacter when detected by PCR only, and subtyped EPEC to see whether this additional testing could give information about their relevance in causing diarrhoea.

## Methods

### Ethical considerations

According to regulations of the regional ethical committee (REK) the study design did not require informed patient consent. The study was presented to and approved by the Internal Privacy Ombudsmann of Akershus University Hospital.

### Design

We conducted a retrospective case-case study. Case groups with 1) EPEC only and 2) EPEC in combination with any other pathogen in the PCR multiplex array, 3) PCR positive/culture negative Campylobacter, and 4) PCR positive/culture positive Campylobacter were compared. Medical records were reviewed and cases classified according to pre-specified clinical criteria as infectious gastroenteritis or non-infectious causes for diarrhoea. We analyzed the association between laboratory findings (the 4 subgroups) and the pre-specified clinical classification. With the further aim of exploring the value of complementary testing, we sequenced culture negative campylobacter and tested EPEC for bundle forming pilus A (bfpA) gene, distinguishing typical from atypical EPEC.

### Setting and patients

Akershus University Hospital is a large secondary referral hospital. We included all hospitalised patients over 18 years of age who had PCR positive stool samples for campylobacter or EPEC from November 2013 to November 2015. We recorded whether confirmatory cultures were positive or negative, along with the type and number of co-infecting pathogens, and whether diarrhoea was documented in the medical records (defined as 3 or more loose stools within 24 h). The records were further reviewed for possible non-infectious causes of diarrhoea including on-going antibiotic or cytostatic therapy, active inflammatory bowel disease (IBD), gastrointestinal tumors, short-bowel syndrome, sepsis, and a miscellaneous group including excessive laxative consumption, hospital admission for alcohol intoxication and severe liver disease. Patients with IBD, who according to medical records had no or little disease activity, were classified as infectious gastroenteritis patients. Pre-specified criteria for unequivocal pathogen related diarrhoeal disease (infectious gastroenteritis) was; abrupt onset, short duration (< 14 days), and no non-infectious causes for diarrhoea identified. Travel related gastroenteritis was defined as diarrhoeal disease occurring within 10 days of returning from travel. We further recorded month of sampling and patient age.

### Microbiological tests

All stool samples were analysed with the help of a PCR based detection panel for intestinal pathogens within 3 days of collection. In addition to EPEC and Campylobacter spp., the panel includes Norovirus, Rotavirus, Adenovirus 40/41, *Giardia lamblia*, Cryptosporidium spp., *Entamoeba histolytica*, *Yersinia enterocolitica*, Salmonella spp., Enterohemorrhagic *Escherichia coli* and Shigella spp. During the study period and for purposes of quality control after the introduction of a PCR based diagnostic, positive samples were also systematically cultured and pathogens identified according to routine microbiological methods. For the PCR assay, nucleic acids were extracted using Qiasymphony DSP Virus/Pathogen Kit (Qiagen) as described before [3]. EPEC and campylobacter were identified with the commercial Ridagene EHEC/EPEC kit and Ridagene Bacterial Stool (R-biopharm) panel respectively which detects *eae* gene and campylobacter *16 s*. For *eae* positive samples, feces samples were cultured on lactose agar at 35 °C. Further in-house PCR typing was performed with probes and primers for *bfpA* [4] and *eae* [5] using Taqman advanced mastermix (Applied biosystems). *Campylobacter jejuni/coli* were cultured using CCDA agar at 42 °C in microaerophilic atmosphere generated by a Campygen system (Oxoid). PCR positive and culture negative campylobacter samples were sequenced using *16 s* primers modified from Platts Mills et al. [6]: Forward: gatgacacttttcggagcgtaa, Reverse: cattgtagcacgtgtgtcgc. Briefly, faecal nucleic acid eluates were amplified with Evagreen plus PCR mix (Takara). Products were sequenced on ABI 3130XL using Bigdye 3.1 reagents (Applied biosystems). Sequences were identified with BLAST [7] and Ripseq [8]. *Clostridium difficile* was tested by ImmunoCard Toxins A&B assay (Meridian bioscience) only when ordered specifically and was thus not part of the PCR panel.

### Statistical methods

Standard descriptive statistics were done with SPSS and Microsoft Excel for Windows. 95% CI for proportions were calculated using the Fisher Exact test and differences between continuous variables were tested with the Student t-test [9].

## Results

In total 291 patients were included, 122 were positive for EPEC of which 38 were co-infected with other gastro intestinal pathogens, 169 were positive for campylobacter of which 55 were culture negative. Twenty-seven patients were labeled as having no diarrhoea when either no mention of diarrhoea was found in the medical records, or the only mention was a single record entry of loose stool in the nurse report, but not in the medical record at admittance and discharge, and the discharge record clearly stated a non-gastro-intestinal diagnosis.These comprised 20% (95% CI 12–30) of the EPEC only group, and 5.3 and 4.7% of the EPEC co-infected and campylobacter group respectively. Clinical characteristics are summarized in Table 1.

**Table 1:** Clinical charateristics in cases with EPEC detected alone, EPEC and other pathogens detected and campylobacter detected

*Multiplex PCR result*	*EPEC detected alone* *N = 84*	*EPEC and other pathogens detected N = 38* ^*b*^	*Campylobacter detected(all)* *N = 169*
Sex (male) [n (%)]	42 (50)	21 (55)	87 (51)
Age years [mean(SD)]	60 (18.6)	55 (19.2)	53 (19.4)
*Other characteristics* [%, (95% CI)]			
Ongoing antibiotic treatment	32 (22–43)	18 (7.7–34)	9.5 (5.5–15)
No diarrhoea	20 (12–30)	5.3 (0.64–18)	4.7 (2.1–9.1)
Ongoing chemotherapy	15 (8.6–25)	2.6 (0.066–14)	3.5 (1.3–7.6)
Travel associated	8.3 (3.4–16)	42 (26–59)	35 (28–43)
Culture positive	49 (38–60)	26 (13–43)	66 (58–73)
bfpA positive patients	4.8 (1.3–12)	5.3 (0.64–18)	–
*Clinical diagnosis* [%, (95% CI)]			
Clinical non-infectious cause of diarrhoea^a^	73 (61–83)	25 (12–42)	17 (11–23)

^a^A total of 27 patients did not present with diarrhoea and are excluded from the analyses

^b^The top three co-infection agents were Campylobacter (*n* = 15), Norovirus (*n* = 9), Salmonella (*n* = 3) and *Clostrium difficile* (*n* = 3)

**Fig. 1:**
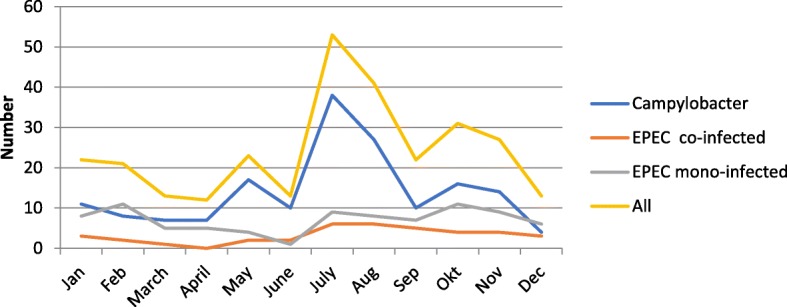
Monthly prevalence of cases with EPEC detected alone, EPEC co-infections and *Campylobacter* detected

A seasonal pattern was seen in the campylobacter group, whereas no seasonal pattern was observed in the EPEC, and EPEC co-infected groups or in the culture negative campylobacter cases (low number of observations per month) (Fig. 1). Patients in the EPEC only group were significantly older (*p* = 0.0066) than in the campylobacter group, 60 (SD 18) years versus 53 (SD 19) years. No age difference was seen between patients in the EPEC co-infected group 55 (SD 19) years and the campylobacter group.

### Patient characteristics in patients with culture positive vs culture negative campylobacter test results

When analysing the Campylobacter PCR positive specimens according to culture outcome, a marked difference in clinical characteristics was observed (Table 2).

**Table 2:** Campylobacter PCR positive-culture negative vs Campylobacter PCR positive-culture positive

*Multiplex PCR result*	*PCR positive-culture negative* *N = 55* ^*b*^	*PCR positive-culture positive* *N = 114*	*Campylobacter detected(all)* *N = 169*
Sex (male) [n (%)]	29 (53)	58 (51)	87 (51)
Age years [mean(SD)]	61 (21)	50 (16)	53 (19)
*Other characteristics* [%, (95% CI)]			
Ongoing antibiotic treatment	24 (13–37)	2.6 (0.5–7.5)	9.5 (5.5–15)
Co-infection	18 (9.1–31)	1.7 (0.21–6.1)	7.1 (3.7–12)
No diarrhoea	9.0 (3.0–20)	0.88 (0.02–4.8)	4.7 (2.7–9.9)
Ongoing chemotherapy	5.4 (1.1–15)	2.6 (0.54–7.5)	3.5 (1.3–7.6)
Travel associated	16 (7.8–29)	45 (35–54)	35 (28–43)
*Clinical diagnosis* [%, (95% CI)]			
Clinical non-infectious cause of diarrhoea	48 (34–63)	4.4 (1.4–10)	17 (11–23)^a^

^a^A total of 6 patients did not present with diarrhoea and are excluded from these analyses

^b^47 of 55 were sequenced as various *Campylobacter* spp., 29 as *Campylobacter concisus* (see Fig. 2)

**Fig. 2:**
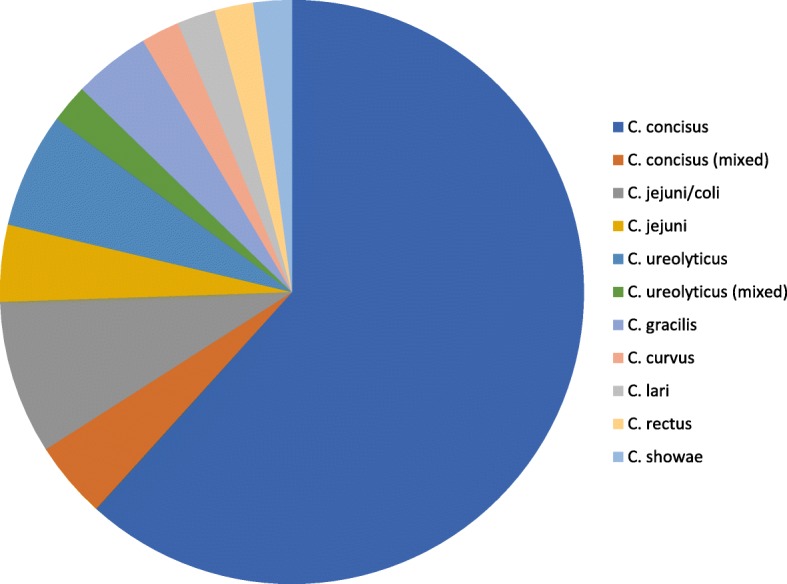
Distribution of sequencing results of 47 out of 55 cases with non-cultivable EPEC PCR positive cases. Twenty-nine were sequenced as *C. concisus*, in additition 2 cases *C. concisus* in mixed infections. *C. jejuni/coli* was the second and *C. ureolyticus* the third most prevalent finding. The remaining spp. were mainly singletons

The culture negative *Campylobacter* patients constituted 31% of total PCR positives and displayed an almost ten-fold lower rate of ongoing antibiotic therapy, clinical non-infectious cause of diarrhoea and no diarrhoea recorded than culture positive patients. Co-infecting pathogens were detected in 18% (95% CI 9. 1–31) of culture negatives. Nearly all culture positives (96% (95% CI 90–99) fulfilled the criteria for clear cut infectious gastroenteritis, and they were in almost all cases detected without other co-infecting pathogens 92% (95% CI 88–92). Sequencing culture negative samples was possible in 47 out of total 55 culture negative samples. *Campylobacter concisus* was identified in 29 samples (62%), other species being mostly singletons (Fig. 2). Six of the *C. concisus* were found in mixed infections (norovirus, Salmonella, Enterohemorrhagic *Escherichia coli* (EHEC)). Only 9/23 (39%) of the *C. concisus* mono-infected fulfilled the criteria for infectious gastroenteritis (data not shown).

### Patient characteristics in patients with EPEC positive test results

The proportion of patients with ongoing antibiotic therapy or chemotherapy was highest in the EPEC only group. The proportion of patients with an alternative non-infectious cause of diarrhoea, was higher in the EPEC only group compared to the EPEC co-infected (*p* = 0.010) and campylobacter group (*p* < 0.0005), 73% (95% CI 61–83) versus 25% (95% CI 12–42) and 17% (95% CI 11–23), respectively. The EPEC co-infected group had the lowest proportion of culture positives, 26% (95% CI 13–43) versus 49% (95% CI 38–60) and 66% (95% CI 58–73) in the EPEC only (*p* = 0.13) and Campylobacter group (*p* = 0.015). The bfpA gene distinguishing typical (tEPEC) from atypical (aEPEC) was found in only 4.8% of EPEC positives.

## Discussion

The main finding of this study was that clinical characteristics differed significantly between culture-positive campylobacter, culture-negative campylobacter, EPEC mono-infected and EPEC co-infected patients. The clinical relevance of these pathogens therefore depends on clinical context. Although there was some overlap among groups, culture positive campylobacter was found almost exclusively in patients whose medical records indicated a clear-cut infectious gastroenteritis, whereas patients with a PCR positive test result only often had an alternative explanation for their diarrhoea. The difference was also reflected in different microbiological findings; Culture positives were almost exclusively *C jejuni/coli*, whereas in culture negatives, constituting a third of the total PCR positives, *C. concisus* was the most frequent species.

The vast majority of EPEC only positives had documented non-infectious factors that could explain diarrhoea and the EPEC only group had the largest proportion of patients with no diarrhoea documented in medical records. This could be due to lack of documentation and not lack of diarrhoea, nevertheless, lack of documentation would not be expected to be biased towards a specific pathogen. The EPEC co-infected group mimicked the culture positive campylobacter group, with most patients fulfilling the infectious gastroenteritis criteria. Of the 38 co-infecting pathogens, 15 were Campylobacter (6 culture positive), 9 were norovirus, 3 were Salmonella (2 culture positive), 2 were Shigella (both culture positive), 3 were *Giardia lamblia* and responded to Giardia specific treatment and 3 patients were positive for *C. difficile* and treated as such (data not shown). As opposed to EPEC, carrier states with these pathogens are usually not found in immuno-competent hosts and argue against EPEC being the principal cause of infectious gastroenteritis in co-infected patients. EPEC and entero aggregative *Escherichia coli* (EAEC) was present in 98 of 116 (84%) of samples with multiple organisms in the European quarterly point-prevalence study of community-acquired diarrhoea (EUCODI), and the authors also raised the question of the clinical relevance of these putative pathogens [10]. Several commercial tests include EPEC in their panels utilising the presence of the eae (evading and effacing) and the absence of the stx (shigatoxin) genes for identification of EPEC. EPEC is subdivided into typical (tEPEC) possessing a virulence plasmid carrying the bfpA gene encoding the bundle-forming pilus (BFP), an established diarrhoeagenic pathogen, and atypical EPEC (aEPEC) lacking this plasmid, for whom pathogenicity is less certain and possibly related to serotypes [11]. In a recent comprehensive review by Hu and Torres, tEPEC was still considered a bona fide pathogen due to their arsenal of virulence factors and association with severe disease. The role of aEPEC as a foe or innocent bystander would in the opinion of the authors require further epidemiological studies elucidating whether certain serotypes are specifically linked to disease in humans [12]. As in other studies from western countries [13] the prevalence of tEPEC expressing bfpA was low (4.8%) in our study, and although three of the four tEPEC positives in this study fell in the infectious gastroenteritis group, testing for bfpA in this low-prevalence population did not help clarify the clinical significance of EPEC.

*C. concisus* has been described as an emerging pathogen [14] and has been linked to inflammatory bowel disease [15], but there are limited clinical data regarding its role in gastroenteritis. For instance, in a case-control study of traveler’s diarrhoea from Nepal and Thailand with stool samples negative for common pathogens, *C. concisus* was identified significantly more often in cases from Nepal (28.9%; 24/83) as compared to controls (4%; 3/75) while detected in only two cases (2/26; 7.7%) and in none of the control stool samples from Thailand [16]. Further studies are needed to establish the role of *C. concisus* in acute infectious gastroenteritis, though our data suggest it is more frequently associated with infectious gastroenteritis than EPEC, *C. concisus* may as EPEC become detectable as the result of gut dysbiosis of other reasons, including antibiotic usage or infections with other gastroenteritis pathogens, as was often the case in our study.

There was a marked seasonal variation for *C jejuni/coli* with a peak incidence in the summer holidays months, as expected, for a pathogen associated with travel, higher temperatures, unrefrigerated food and outdoor cooking. This seasonality was not seen for EPEC, EPEC co-infected and *C. concisus* cases, but the absolute number of cases per month in our study is low. The higher age of EPEC patients may reflect a higher non-gastroenteritis related morbidity with increasing age, and seen together with a lack of seasonality, this may strengthen the view of *C. concisus* and EPEC as innocent bystanders.

The strength of this study is the linkage of clinical and microbiological data, as diagnostic testing of fecal sampling is often limited by use of an inappropriate comparative standard, i.e. standard other than clinical disease. Detecting cases through the microbiological department database secured complete registration and inclusion over a long period of time. Criteria for non-infectious diarrhoea were pre-specified and clearly defined and medical records were consistently reviewed by one experienced clinician blinded for culture results. Further, the use of antibiotic or cytostatic medication is an objective variable. Our study has limitations; due to its retrospective nature misclassifications and omissions in the medical records cannot be ruled out, wrongly attributing pathogen related diarrhoea to non-infectious related causes or vice versa. However, the misclassification should be non-differential and bias should be towards the null. The large difference in rates of clear-cut pathogen related gastroenteritis and non-infectious related diarrhoeagenic conditions between the EPEC mono-infected and the other two groups seem valid. By design, a positive pathogen finding was the inclusion criteria, not a new diarrhoeal episode. Thus, clinical and microbiological findings in patients with diarrhoea for whom no fecal sample was taken remain unknown, as well as clinical findings in patients tested but with no microbiological findings. The low incidence of *C. difficile* co-infections may seem unexpected, as many diarrhoea patients were on antibiotic or cytotstatic therapy. However, *C. difficile* tests are ordered separately and are not part of the faeces PCR platform. With our case-finding strategy, patients with *C. difficile* diarrhoea due to antibiotic therapy will not be identified if fecal testing is done correctly, that is testing only for *C. difficile* and not adding faeces PCR for other gastroenteritis pathogens. The few co-infections with *C. difficile* were identified through reading of the medical records of the faeces PCR positives, and not through searches in the microbiology database. Cultures for *Campylobacter jejuni/coli* were performed at 42 °C in microaerophilic atmosphere, which is our standard cultivation method, and primarily aimed at detecting *C. jejuni, C. coli* and *C. lari*, the non-thermophilic species considered to cause only a minor proportion of human disease. Non-thermophilic campylobacters such as *C. concius* might thus have been missed. The study aim was, however, to present and discuss microbiological findings when using standard culture methods. The conclusion of far more non-diarrhoeagenic conditions in campylobacter culture negative patients and conversely far more clear-cut gastroenteritis patients in the culture positive patients thus remain valid. Cultivating methods suitable for the non-thermophilic campylobacter should be considered in future study settings as the role of *C. concisus* is unsettled. Finally, the study is limited to hospitalised patients with positive PCR test results, and the results should not be extrapolated to other patient groups.

## Conclusions

Campylobacter detected by PCR only differed from culture positive samples. The majority of the former were *C. concisus,* and the latter *C. jejuni/coli*. This difference in species distribution was reflected in the prevalence of non-infectious causes of diarrhoea. Culture negatives had almost ten-fold higher rate of ongoing antibiotic therapy, clinical non-infectious cause of diarrhoea, co-infections, and no diarrhoea documented than culture positives, which fulfilled almost exclusively the infectious gastroenteritis criteria. Conditions associated with diarrhoea other than infectious gastroenteritis was found in the majority of EPEC only positive patients. In hospitalised patients with diarrhoea the relevance of identifying culture negative campylobacter and EPEC must be critically evaluated, and other diarrhoeagenic conditions carefully considered.

## Data Availability

The datasets generated and/or analysed during the current study are not publicly available due regulation from the Internal Privacy Ombudsmann of Akershus University Hospital, but are kept on a secure hospital server and are available from the corresponding author on reasonable request.

## Abbreviations

bfpA: bundle forming pilus A
EHEC: Enterohemorrhagic *Escherichia coli*
EPEC: Enteropathogenic *Escherichia coli*
IBD: inflammatory bowel disease
PCR: Polymerase Chain Reaction
